# Supplementary material for: The Effect of Botulinum Neurotoxin-A (BoNT-A) on Muscle Strength in Adult-Onset Neurological Conditions with Focal Muscle Spasticity: A Systematic Review
Source: Toxins (Basel). 2024 Aug 8;16(8):347. doi: 10.3390/toxins16080347 (PMC11359732; doi:10.3390/toxins16080347)
Supplement: Supplementary file 1 [file toxins-16-00347-s001.zip › Supplementary Table S1. Agonist Strength Outcomes from articles included in the analysis - Revised.pdf]

Supplementary Table S1. Agonist strength outcomes from articles included in the analysis (n = 13).

Study	Outcome Measure (Unit Of Measure)	Group Details/Design	Muscle Or Movement	Pre-Injection, Mean, SD Or [IQR] (95% CI)	Post-Injection Mean, SD (95% CI) Median (P25, P75) Min, Max	Within-Group Difference, Mean +/- SD. Median (P25, P75) Min, Max	p Value (Within-Group Change)	Within-Group Change			Timepoints
								Significantly Improved	Unchanged	Significantly Worsened	
Bernuz 2012 [19]	Peak Voluntary Torque 60°/s Hip 90° (Nm)	Pre-Post	Quadriceps	63.585 (36.085) ^	54.015 (30.117) ^	NR	0.0004 (Combined Result)<br><br>(p< 0.001) Is SS		✓ ƒ		T1 = 4-6/52
	Peak Voluntary Torque 60°/s Hip 0° (Nm)		Quadriceps	41.83 (33.30) ^	39.49 (29.97) ^				✓ ƒ		
Bollens 2013 [46]	MRC (0-5)<br>*Median (IQR)	BoNT-A Alone	Triceps Surae	2.5 (1.5/3)	2.5 (2/4) T1<br>2.5 (1.5/3.5) T2	0 (0/1)<br>0 (0/0)	0.279<br>0.328		✓<br>✓		T1 = 2/12<br>T2 = 6/12
Chen 2020 [69]	MVC (Nm)<br>*Mean ± SD	Pre-Post	Elbow Flexion	12.73 ± 6.37	9.98 ± 4.64 T1	NR	<0.05			✓	T1 = 3/52
Chen 2022 [68]	MVC (Nm)<br>*Mean ± SD	Pre-Post	Elbow Flexion	12.73 ± 6.37	13.37 ± 4.23 T2<br>T2 - Data From 2022 Study)	NR	0.02 (T0-T2)		✓ (T0-T2)		T2 = 3/12
Cinone 2019 [32]	Peak Torque – Concentric Isokinetic Dynamometer MVC Angular Rate Of 60°/s (Nm)<br>*Mean (SD)	BoNT-A + 4/52 Isokinetic Training	Plantarflexion	10.09 (0.63)	9.73 (0.52) T1<br>9.85 (0.66) T2	NR	0.005 (T0-T1)<br>NR		 ✓	✓	T1 = 5/52<br>T2 = 8/52
		BoNT-A Alone		10.09 (0.63)	8.6 (0.80) T1<br>8.97 (1.0) T2	NR	0.042 (T0-T1)<br>0.032 (T0-T2)			✓<br>✓	
de Niet 2015 [70]	MRC (0-5)<br>*Median [IQR]	Pre-Post	Gastrocnemius + Soleus	5 [4–5]	4 [4–5] T1<br>5 [4–5] T2	NR	0.046 (T0-T1)<br>0.317 (T0-T2)		 ✓	✓	T1 = 4/52<br>T2 = 18/52
	QMA - Myometry Testing System (Kg)<br>*Mean (SD) [95% CI]		Gastrocnemius + Soleus	52.6 (18.1) [43.5–61.8]	50.5 (15.9) [42.6–58.5] T1<br>53.6 (15.8) [45.7–61.6] T2	NR	NR<br>0.190 (T0 – T2)		✓		
Diniz de lima 2021 [48]	MRC (0-5)<br>*Mean ± SD	Pre-Post Cross Over Trial (BoNT-	Hip Adductors	4.84 ± 0.46	4.55 ± 0.77	NR	0.10		✓		T1 = 8/52
			Triceps Surae	4.94 ± 0.30	4.95 ± 0.20	NR	0.99		✓		

Supplementary Table S1. Agonist muscle strength outcomes from articles included in the analysis.

		A Phase Only)									
Hameau 2014 [33]	Peak Concentric Torque – MVC Constant Angular Velocities (Nm)	Pre-Post	Knee Extension<br>30°/S<br>60°/S<br>90°/S	54.5 ± 25.9 ^<br>49.9 ± 22.7 ^<br>45.1 ± 21.9 ^	43.1 ± 24.3 ^<br>39.5 ± 23.0 ^<br>36.9 ± 20.1^	NR	0.009<br>0.016<br>0.015			✓<br>✓<br>✓	T1 = 1/12
	Peak Isometric Torque - MVC Constant Angular Velocities (Nm)		Knee Extension<br>40°<br>60°	76.3 ± 28.0^<br>73.4 ± 25.8^	65.3 ± 26.8 ^<br>63.4 ± 26.2 ^	NR	0.004<br>0.015			✓<br>✓	
Lee 2018 [75]	MRC (0-5)<br>*Mean ± SD	Pre-Post	Finger Flexor	2.60 ± 0.51	2.60 ± 0.51 T1<br>2.67 ± 0.49 T2	NR	1.000 T0-T1<br>0.317 T1-T2<br>0.564 T0-T2<br>0.717 (Overall)		✓<br>✓<br>✓		T1 = 2/52<br>T2 = 6/52
Lim 2016 [90]	MRC (0-5)<br>*Mean ± SD	Subacute	Elbow Flexor	3.00 ± 0.50	3.33 ± 0.71	NR	0.083		✓		T1 = 4/52
			Wrist Flexor	2.33 ± 1.32	2.67 ± 1.12	NR	0.180		✓		
		Chronic	Elbow Flexor	3.22 ± 0.67	3.44 ± 0.53	NR	0.157		✓		
			Wrist Flexor	1.56 ± 1.33	1.56 ± 1.33	NR	1.000		✓		
Pandyan 2002 [30]	Isometric Muscle Strength (N)<br>*Mean (SD)	Pre-Post	Elbow Flexion	35.24 (13.71) ^ *f*	31.56 (14.70) ^ *f*	– 3.68 ± 16.15 ^ *f*	NR		✓		T1 = 4/52
Rousseaux 2002 [82]	MRC (0-5)<br>*Mean (SD)	Pre-Post	Wrist Flexors	2.45 (1.21)	2.50 (1.19) T1<br>2.55 (1.22) T2<br>2.30 (1.22) T3	0.05 (0.28)<br>0.10 (0.31)<br>– 0.15 (0.37)	NR<br>NR<br>0.013 ‡		NR<br>NR	✓ (T3)	T1 = 2/52<br>T2 = 2/12<br>T3 = 5/12
Rousseaux 2005 [81]	MRC (0-5)<br>*Mean (SD)	Pre-Post	Ankle Plantarflexors	2.76 (0.95)	2.76 (0.99)<br>2.77 (0.96)<br>2.78 (1.00)	0.00 (0.21)<br>0.01 (0.22)<br>0.02 (0.29)	NR<br>NR<br>0.773		NR<br>NR<br>✓ (T3)		T1 = 2/52<br>T2 = 2/12<br>T3 = 5/12

f - Calculated By Authors Based On Supplied Data, ^ - Data Supplied By Authors Upon Request, ‡ - The p-value represents the significance of the influence of factor evaluation (D1, D15, M2, M5) on each dependent variable, HHD – Handheld Dynamometer, Kg – Kilograms, MMT – Manual Muscle Test, MRC – Medical Council Research Scale (0-5), MVC – Maximal Voluntary Contraction, MVP – Maximal Voluntary Power Dynamometer, N – Newtons, Nm – Newton Metres, NR – Not Reported Significance Result, NS - Not Significant, PF- Plantarflexors, QMA – Quantitative Muscle Assessment- Fixed Myometry Muscle Testing, s- seconds, SD – Standard Deviation, SS - Statistically Significant.
Significance is reported as $p<0.05$ unless otherwise stated.

Supplementary Table S1. Agonist muscle strength outcomes from articles included in the analysis.
