# Supplementary material for: The Effect of Botulinum Neurotoxin-A (BoNT-A) on Muscle Strength in Adult-Onset Neurological Conditions with Focal Muscle Spasticity: A Systematic Review
Source: Toxins (Basel). 2024 Aug 8;16(8):347. doi: 10.3390/toxins16080347 (PMC11359732; doi:10.3390/toxins16080347)
Supplement: Supplementary file 1 [file toxins-16-00347-s001.zip › Supplementary Table S2. Antagonist Strength Outcomes from articles included in the analysis - Revised.pdf]

Supplementary Table S2. Antagonist strength outcomes from articles included in the analysis (n = 11).

Study	Outcome Measure (Unit of Measure)	Group Details/Design	Muscle or Movement	Pre-Injection Mean, SD (Or 95% CI)	Post-Injection Mean, SD (95%CI) Median (P25, P75) Min, Max	Within-Group Difference, Mean +/- SD. Median (P25,P75) Min/Max	p Value (Within-Group Change)	Within-Group Change			Timepoints
								Significantly improved	Unchanged	Significantly worsened	
Baricich 2019 [45]	MRC (0-5)	G1: ES of Injected Muscles + ES of TA (n=15)	Tibialis Anterior	G1: 1.0 (0.0)	1.0(1.0)<br>2.0 (1.0)<br>1.0 (1.0)	NR	0.269 - T0-T1<br>0.143 - T0-T2<br>0.269 - T0-T3		✓<br>✓<br>✓		T1 = 10/7<br>T2 = 20/7<br>T3 = 90/7
		G2: ES of Injected Muscles (n=15)		G2: 1.0 (0.0)	1.0 (1.0)<br>2.0 (1.0)<br>1.0 (1.0)	NR	0.999 - T0-T1<br>0.472 - T0-T2<br>0.999 - T0-T3		✓<br>✓<br>✓		
Bollens 2013 [46]	MRC (0-5)<br>*Median (IQR)	BoNT-A Only	Tibialis Anterior	3.0 (0.5/4)	2.0 (0/4) T1<br>1.0 (0/4) T2	NR	0.442<br>0.161<br>(Mann Whitney Test)		✓<br>✓		T1 = 2/12<br>T2 = 6/12
Carda 2011 [47]	MRC (0-5)	Taping (n=24)	Tibialis Anterior	1.1 ± 0.7	2.0 ± 0.8<br>1.9 ± 0.6	NR	All NS as per Table 2 Key<br><br>p Value Set At <0.02		✓<br>✓		T1 = ~3/52<br>T2 = 3/12
		Casting (n=27)		1.9 ± 0.5	2.8 ± 0.6<br>2.7 ± 0.8	NR			✓<br>✓		
		Stretching (n=18)		0.9 ± 0.3	1.7 ± 0.8<br>1.6 ± 0.9	NR			✓<br>✓		
Cinone 2019 [32]	Isokinetic Peak Torque 60°/s (Nm)<br>*Mean (SD)	BoNT-A + 4/52 Isokinetic Training	Dorsiflexion	8.6 (0.15)	10.82 (1.59)<br>9.93 (1.45)	NR	p< 0.05<br>p< 0.05	✓<br>✓			T1 = 5/52<br>T2 = 8/52
		BoNT-A Alone		8.91 (0.82)	9.71 (0.92)<br>9.28 (0.95)	NR	p<0.05<br>NR	✓	 ✓		
Hameau 2014 [33]	MVC Peak Concentric Torque (Nm)	Pre-Post	Knee Flexion<br>30°/s<br>60°/s<br>90°/s	21.8 ± 20.0 ^<br>16.4 ± 14.3 ^<br>16.5 ± 13.1 ^	27.3 ± 23.0 ^<br>22.2 ± 18.0 ^<br>21.0 ± 12.7 ^	NR	0.015<br>0.003<br>NS	✓<br>✓	 <br>✓		T1 = 1/12
	MVC Peak Isometric Torque (Nm)		Knee Flexion<br>40°<br>60°	34.8 ± 24.5 ^<br>24.0 ± 17.8 ^	41.6 ± 28.1 ^<br>27.6 ± 19.0 ^	NR	0.011<br>0.041	✓<br>✓			T1 = 1/12
Lee 2018 [75]	MRC (0-5)<br>*Mean ± SD	Pre-Post	Finger Extensor	1.47 ± 0.64	1.60 ± 0.63 T1<br>1.67 ± 0.62 T2	NR<br>NR	0.157 T0-T1<br>0.083 T0-T2		✓<br>✓		T1–T2 = 2/52<br>T2–T3 = 2-6/52

Supplementary Table S2. Antagonist outcomes from articles included in the analysis.

							0.097<br>(Friedman<br>Test)				T1–T3 = 0-6/52
Leung 2019 [54]	MRC (0-5)	BoNT-A +<br>Serial Casting	Dorsiflexion<br>Median (IQR)	0 (0–3) (n=7)	1(0-1) – T1<br>(n=7)<br>1(0-4) – T2<br>(n=7)	NR	NR		T1: ✓ ~2-4/52<br>T2: ✓ ~4-8/52<br>T3: ✓ ~10-<br>12/52		T1 = Measured<br>at Time Post-<br>Injection 5 Days<br>+ ∑ 27 Days<br>(IQR: 15–31<br>Days)<br>~2-4/52<br>T2 = 2/52 Post<br>Cast + ∑ 27 Days<br>(IQR: 15–31<br>Days)<br>= ~4-8/52<br>T3 = 8/52 Post<br>Cast + ∑ 27 Days<br>(IQR: 15–31<br>Days)<br>=~10 – 12/52
		BoNT- A +<br>Waitlist 6/52 +<br>Serial Casting		0 (0–2) (n=6)	3 (0-4) – T1<br>(n=7)<br>3 (0-4) – T2<br>(n=6)	NR	NR		T1: ✓ ~2-4/52<br>T2: ✓ ~4-8/52<br>T3: ✓ ~10-<br>12/52		
		Total (of the<br>above two<br>groups)		0 (0–3) (n=13)	1 (1–1) T1<br>(n=13)<br>1 (1–4) T2<br>(n=12)<br>2 (1–4) T3<br>(n=12)	Z = −1.27<br>Z = −1.40<br>Z = −1.69	0.21 T1<br>0.16 T2<br>0.09 T3		T1: ✓ ~2-4/52<br>T2: ✓ ~4-8/52<br>T3: ✓ ~10-<br>12/52		
Lim 2016 [90]	MRC (0-5)	Subacute	Elbow<br>Extensor	2.11 ± 1.27	3.11 ± 0.93	NR	0.014<br>(p<0.05<br>Wilcoxon<br>Signed-Rank<br>test within<br>each group)	✓			T1 = 4/52
			Wrist Extensor	2.22 ± 1.30	2.33 ± 1.41	NR	0.317		✓		
		Chronic	Elbow<br>Extensor	2.56 ± 0.73	2.78 ± 0.44	NR	0.157		✓		
			Wrist Extensor	1.22 ± 1.09	1.44 ± 1.01	NR	0.157		✓		
Pandyan 2002<br>[30]	Isometric<br>Muscle<br>Strength (N)<br>*Mean	Pre-Post	Elbow<br>Extension	16.24 (18.69) ^<br>*ƒ*	18.87 (18.14) *ƒ*	2.63 (24.82) ^ *ƒ*	NR		✓		T1 = 4/52
Rousseaux 2002<br>[82]	MRC (0-5)<br>*Mean (SD)	Pre-Post	Wrist<br>Extensors	1.88 (1.09)	2.18 (1.20) T1<br>2.03 (1.21) T2<br>1.98 (1.23) T3	0.30 (0.41)<br>0.15 (0.24)<br>0.10 (0.38)	NR<br>NR<br>0.008 ‡	✓ (T3)	NR<br>NR		T1 = 2/52<br>T2 = 2/12<br>T3 = 5/12
			Finger<br>Extensors	1.53 (1.20)<br>(Day of<br>Injection)	1.65 (1.20) T1<br>1.68 (1.23) T2<br>1.73 (1.27) T3	0.13 (0.28)<br>0.15 (0.29)<br>0.20 (0.25)	NR<br>NR<br>0.013 ‡	✓ (T3)	NR<br>NR		
Rousseaux 2007<br>[83]	MRC (0-5)<br>*Median	Pre-Post	Ankle<br>Dorsiflexion<br>Right Leg	2	2 T1<br>2 T2<br>2 T3	NR	0.265	NS (T3)	NR<br>NR		T1 = 2–3/52<br>T2 = 2–3/12<br>T3 = 5/12

Supplementary Table S2. Antagonist outcomes from articles included in the analysis.

			Ankle	2	2 T1	NR			NR		
			Dorsiflexion		2 T2				NR		
			Left Leg		2 T3		0.056	NS (T3)			

ƒ - Calculated By Authors Based On Supplied Data, ^ - Data supplied by author upon request, ‡ - The p-value represents the significance of the influence of factor evaluation (D1, D15, M2, M5) on each dependent variable, ES – E-Stims, HHD – Handheld Dynamometer, Kg – Kilograms, MMT – Manual Muscle Test, MRC – Medical Council Research Scale (0-5), MS – Muscle Strength, MVC – Maximal Voluntary Contraction, MVP – Maximal Voluntary Power Dynamometer, Nm – Newton Metres, N – Newtons, NR – Not Reported Significance Result, NS - Not Significant, PF- Plantarflexors, QMA – Quantitative Muscle Assessment - Fixed Myometry Muscle Testing, s – second, SD – Standard Deviation, SS - Statistically Significant.

Significance is reported as $p<0.05$ unless otherwise stated.

Supplementary Table S2. Antagonist outcomes from articles included in the analysis.
