# Supplementary material for: The Effect of Botulinum Neurotoxin-A (BoNT-A) on Muscle Strength in Adult-Onset Neurological Conditions with Focal Muscle Spasticity: A Systematic Review
Source: Toxins (Basel). 2024 Aug 8;16(8):347. doi: 10.3390/toxins16080347 (PMC11359732; doi:10.3390/toxins16080347)
Supplement: Supplementary file 1 [file toxins-16-00347-s001.zip › Supplementary Table S3. Global Strength Outcomes from articles included in the analysis - Revised.pdf]

Supplementary Table S3. Global strength outcomes from articles included in the analysis (n = 6).

Study	Outcome Measure (Unit Of Measure)	Group Details/Design	Muscle or Movement	Pre-injection, Mean, SD or [IQR] (95% CI)	Post-injection Mean, SD (95% CI)<br>Median (P25, P75)<br>Min, Max	Within-Group Difference, Mean +/- SD.<br>Median (P25, P75)<br>Min, Max	p Value (Within-Group Change)	Within-Group Change			Timepoints
								Significantly improved	Unchanged	Significantly worsened	
Cinone 2019 [32]	Motricity Index (Total out of 100)<br>*Mean ± SD	BoNT-A + 4/52 Isokinetic Training	Lower Extremities	51.67 ± 10.12	54.17 ± 8.65 T1<br>54.17 ± 9.65 T2	NR	NR		NS<br>NS		T1 = 5/52<br>T2 = 8/52
		BoNT-A Alone		49.18 ± 10.25	52.18 ± 10.25 T1<br>53.18 ± 12.09 T2	NR	NR		NS<br>NS		
Giray 2020 [44]	Motricity Index (Total out of 100)	BoNT-A + Lycra Sleeve Plus Rehabilitation	Upper Limb	50 (33.81, 60.58)	67 (53.94,76.05) T1<br>64.5 (51.09,72.5) T2	NR	<0.05 (T0-T1)<br><0.05 (T0-T2)	✓<br>✓			T1 = 3/52<br>T2 = 3/12
		BoNT-A + Rehabilitation Only Group		44.5 (34.34, 56.85)	68 (46.86, 73.33) T1<br>60 (44.64 -70.95) T2		<0.05 (T0-T1)<br><0.05 (T0-T2)<br>* p <0.05 by Wilcoxon test.	✓<br>✓			
Lannin 2020 [53]	Grip strength (Kg)<br>HHD	BoNT-A + Evidence-Based Movement Training	Grip	3.6 (4.3) (n=69)	4.6 (5.7) (n=67)	1.0 (3.4)	NR		NS		T1 = 3/12
		BoNT-A + Usual Care		4.7 (5.9) (n=71)	4.1 (4.4) (n=71)	- 0.4 (3.9)			NS		
Lannin 2022 [52]	Grip strength (Kg)<br>HHD	BoNT-A + Evidence-Based Movement Training		3.6 (4.3) (n=69)	3.8 (4.9) (n=64/65)	0.2 (3.0)	NR		NS		T1 = 12/12
		BoNT-A + Usual Care 3/12 Intense Program		4.7 (5.9)	4.1 (4.8) (n=66/68)	-0.1 (4.5)			NS		
		All Participants		4.0 (5.1) (n=133)	4.0 (4.9) (n=133)	0.1 (-0.6 to 0.7)	NR		NS		
Lee 2018 [75]	Grip Strength (Kg)<br>HHD<br>*Mean ± SD	Pre-Post	Grip	0.27 ± 1.03	0.13 ± 0.52 T1<br>0.33 ± 1.05 T2		0.317 T0-T1<br>0.317 T0-T2<br>0.180 T2-T3<br>0.223 (Overall)		NS<br>NS		T1 = 2/52<br>T2 = 6/52
Pandyan 2002 [30]	GSM (N)<br>*Mean	Pre-post	Grip	14.31 (11.74) ^ ƒ	18.08 (16.42) ^ ƒ	3.77 (10.67) ^ ƒ	0.23 ^ ƒ		NS		T1= 4/52

Supplementary Table S3. Gross Strength Outcomes from articles included in the analysis.

ƒ - Calculated By Authors Based On Supplied Data, ^ - Data Supplied by Author Upon Request, HHD – Handheld Dynamometer, GSM – Grip Strength Meter, MRC – Medical Council Research Scale (0-5), MVC – Maximal Voluntary Contraction, MVP – Maximal Voluntary Power Dynamometer, N- Newtons, Nm- Newton Metres, NR – Not Reported Significance Result, NS - Not Significant, SD – Standard Deviation, SS - Statistically Significant

Significance is reported as $p<0.05$ unless otherwise stated.

Supplementary Table S3. Gross Strength Outcomes from articles included in the analysis.
