# Supplementary material for: The Effect of Botulinum Neurotoxin-A (BoNT-A) on Muscle Strength in Adult-Onset Neurological Conditions with Focal Muscle Spasticity: A Systematic Review
Source: Toxins (Basel). 2024 Aug 8;16(8):347. doi: 10.3390/toxins16080347 (PMC11359732; doi:10.3390/toxins16080347)
Supplement: Supplementary file 1 [file toxins-16-00347-s001.zip › Supplementary Table S4. Spasticity Outcomes from articles included in the analysis - Revised.pdf]

# Supplementary Table S4. Spasticity outcomes from articles included in the analysis (n = 12).

| Study | Outcome Measure (Unit Of Measure) | Group Details/Design | Muscle or Movement | Pre-Injection, Mean, SD (Or 95% CI) | Post-Injection Mean, SD (95%CI) Median (P25, P75) Min, Max | Within-Group Difference, Mean +/- SD. Median (P25, P75) Min/Max | p Value (Within-Group Change) | Within-Group Change | | | Timepoints |
|---|---|---|---|---|---|---|---|---|---|---|---|
| | | | | | | | | Significantly improved | Unchanged | Significantly worsened | |
| Bernuz 2012 [19] | MTS (0 – 4) | Pre-Post | Quadriceps | 2.7 ± 0.7 | 1.9 ± 1 | NR | < 0.05 | ✓ | | | T1 = 4-6/52 |
| | MTS Angle ° | | Quadriceps | 50° ± 30° | 93° ± 44° | NR | < 0.01 | ✓ | | | |
| | Peak Spastic Torque – (Nm) Velocity 90 °/s, Hip 90° | | Quadriceps | 6.7 ± 1.9 | 5.6<br>5.5 | NR | 0.03 | ✓ | | | |
| | Peak Spastic Torque – (Nm) Velocity 150 °/s, Hip 90° | | | 12.2 ± 3.3 | 10.2 ± 6.4 | NR | 0.47 | | ✓ | | |
| | Peak Spastic Torque – (Nm) Velocity 90 °/s, Hip 0° | | | 12.3° ± 9° | + 5 ° | NR | 0.03 | ✓ | | | |
| | Peak Spastic Torque – (Nm) Velocity 150 °/s, Hip 0° | | | 22.4 ± 18.8 | + 4 ° | NR | 0.08 | | ✓ | | |
| Bollens 2013 [46] | MAS (0 – 4) | Pre-Post | Triceps Surae - KE | 3 (2/3.5) | 2 (0.5/3)<br>2.5 (1/3.5) | NR | NR<br>0.236 | | NR<br>✓ | | T1 = 2/12<br>T2 = 6/12 |
| | MAS (0 - 4) | | Soleus - KF | 2.5 (2/3) | 1 (1/2)<br>2 (0.5/2) | NR | NR<br>0.050 | <br>✓ | NR | | |
| | Tardieu (0 -4) | | Soleus - KF | 3 (3/4) | 2 (0.5/2)<br>2.5 (1/3) | NR | NR<br>0.018 | <br>✓ | NR | | |
| Chen 2020 [69] | MAS (0-5) | Pre-Post | Elbow Flexor | 2.2 (0.42) ƒ | 2.2 (0.6) ƒ | 0.00 ± 0.47 ƒ | < 0.05 | ✓ | | | T1 = 3-4/52 |
| | Reflex Torque (Nm) | | | 3.37 ± 2.25 | 1.91 ± 1.35 | 40.8 ± 27.7% | <0.01 | ✓ | | | |
| Chen 2022 [68] | MAS (0-5) | Pre-Post | Elbow Flexor | 3.08 (0.29) ƒ | 2.25 (0.62) ƒ<br>2.75 (0.45) ƒ | -0.83 ± 0.58 ƒ<br>-0.33 ± 0.49 ƒ | <0.01<br><0.05 | ✓<br>✓ | | | T1 = 3/52<br>T2 = 3/12 |
| | Reflex Torque (Nm) | | | 3.48 ± 2.05 | 2.26 ± 1.47<br>3.14 ± 2.04 | NR | <0.01 (T0-T1)<br>0.05 (T0-T2) | ✓ | <br>✓ | | |

Supplementary Table S4. Spasticity Outcomes from articles included in the analysis.

| | | | | | | | | | | | |
|---|---|---|---|---|---|---|---|---|---|---|---|
| Cinone 2019 [32] | MAS (0-4)<br>Median | BoNT-A + 4/52 Isokinetic Training | Gastrocnemius | 2 | 1+<br>1+ | NR | NR<br>NR | | ✓<br>✓ | | T1 = 5/52<br>T2 = 8/52 |
| | | BoNT-A Alone | | 2 | 1+<br>1+ | NR | NR | | ✓<br>✓ | | |
| | MAS (0-4)<br>Median | BoNT-A + 4/52 Isokinetic Training | Soleus | 2 | 1+<br>1+ | NR | NR<br>NR | | ✓<br>✓ | | |
| | | BoNT-A Alone | | 2 | 1+<br>1+ | NR | NR | | ✓<br>✓ | | |
| | Tardieu<br>Median/Mean (SD) | BoNT-A + 4/52 Isokinetic Training | Gastrocnemius | 19.45 (5.8) | 13.45 (4.8)<br>15.78 (5.8) | NR | <0.05<br>NR | ✓ | <br>✓ | | |
| | | BoNT-A Alone | | 20 (4.6) | 14.25 (6.6)<br>16.45 (7.2) | NR | <0.05<br>NR | ✓ | <br>✓ | | |
| | Tardieu<br>Median/Mean (SD) | BoNT-A + 4/52 Isokinetic Training | Soleus | 13.05 (6.6) | 9.45 (5.8)<br>9.03 (5.8) | NR | <0.05<br><0.05 | ✓<br>✓ | | | |
| | | BoNT-A Alone | | 15 (5.9) | 10.76 (5.4)<br>11.91 (4.56) | NR | <0.05<br>NR | ✓ | <br>✓ | | |
| | Peak Resistive Torque 10 Deg/S (Nm) | BoNT-A + 4/52 Isokinetic Training | Plantarflexors | 2.35 (0.65) | 2.31 (0.78)<br>2.32 (0.76) | NR | NR<br>NR | | ✓<br>✓ | | |
| | | BoNT-A Alone | | 2.41 (0.62) | 2.35 (0.54)<br>2.36 (0.79) | NR | NR | | ✓<br>✓ | | |
| | Peak Resistive Torque 30 Deg/S (Nm) | BoNT-A + 4/52 Isokinetic Training | | 3.25 (1.14) | 3.24 (0.5)<br>3.28 (0.81) | NR | NR<br>NR | | ✓<br>✓ | | |
| | | BoNT-A Alone | | 3.12 (1.12) | 3.09 (0.95)<br>3.10 (0.12) | NR | NR<br>NR | | ✓<br>✓ | | |
| | Peak Resistive Torque 90 Deg/S (Nm) | BoNT-A + 4/52 Isokinetic Training | | 9.1 (1.13) | 8.31 (1.2)<br>8.5 (1.17) | NR | <0.05<br><0.05 | ✓<br>✓ | | | |
| | | BoNT-A Alone | | 9.35 (1.25) | 8.75 (0.97)<br>9.2 (1.25) | NR | 0.046<br>NR | ✓ | <br>✓ | | |
| | Peak Resistive Torque 180 Deg/S (Nm) | BoNT-A + 4/52 Isokinetic Training | | 11.7 (1.88) | 10.9 (1.59)<br>10.91 (1.12) | NR | <0.05<br><0.05 | ✓<br>✓ | | | |
| | | BoNT-A Alone | | 12.1 (1.79) | 11.40 (1.35)<br>11.7 (1.12) | NR | <0.05<br>NR | ✓ | <br>✓ | | |
| de Niet 2015 [70] | MAS (0-5) | Pre-Post | Gastrocnemius | 1 (1-2) | 0 (0-0)<br>1 (1-1) | NR | NR<br>NR<br><0.001 | <br>✓(T1-T2) | <br><br>NR | | T1 = 4/52<br>T2 = 18/52 |
| | | | Soleus | 2 (1-2) | 0 (0-1)<br>1 (1-1) | NR | NR<br>NR<br><0.001 | ✓ | | | |

Supplementary Table S4. Spasticity Outcomes from articles included in the analysis.

| | | | | | | | | | | | |
|---|---|---|---|---|---|---|---|---|---|---|---|
| Diniz de lima 2021 [48] | MAS (0-4) | Pre-Post Cross Over Trial (BoNT-A Phase Only) | Hip Adductors | 1.47 ± 0.72 | 1.12 ± 0.74 | NR | NR | | NR | | T1 = 8/52 |
| | | | Triceps Surae | 1.71 ± 0.82 | 1.55 ± 0.88 | NR | NR | | NR | | |
| Hameau 2014 [33] | Peak Resistive Torque Nm at 10 deg/s | Pre-Post | Rectus Femoris | 2.31 (0.68) | 2.26 (0.69) | NR | NS | | ✓ | | T1 = 4/52 |
| | Peak Resistive Torque Nm at 90 deg/s | | | 9.10 (1.14) | 8.31 (1.63) | NR | 0.007 | ✓ | | | |
| | Peak Resistive Torque Nm at 180 deg/s | | | 11.43 (1.89) | 10.39 (2.59) | NR | 0.035 | ✓ | | | |
| | Peak Resistive Torque Nm at 240 deg/s | | | 12.68 (1.85) | 11.25 (2.62) | NR | 0.023 | ✓ | | | |
| | Angle at Peak Torque ° at 90 deg/ s | | | 69.55° (8.62) | 75.07° (4.34) | NR | 0.009 | ✓ | | | |
| | Angle at Peak Torque ° at 180 deg/s | | | 72.79° (5.32) | 77.97° (3.08) | NR | 0.009 | ✓ | | | |
| | Angle At Peak Torque ° at 240 deg/s | | | 74.21° (3.68) | 78.15° (3.42) | NR | 0.013 | ✓ | | | |
| | MAS (0-4) *Mean | | | 1.89 (0.74) | 1.54 (0.60) | NR | 0.027 | ✓ | | | |
| | Duncan Ely ° | | | 61.15 (23.64) | 70.76 (25.23) | NR | 0.092 | | ✓ | | |
| Lee 2018 [75] | MAS (0-4) | Pre-Post | Finger Flexion | 2.33 ± 0.49 | 1.67 ± 0.49<br>1.60 ±- 0.51 | NR<br>NR | 0.002<br>0.317<br>0.001<br>< 0.001 | ✓(T0-T2) | ✓ T0-T1<br>✓ T1-T2<br>✓ T2-T3 | | T1 = 2/52<br>T2 = 6/52 |
| Lim 2016 [90] | MAS (0-5) | Subacute | Elbow Extension (Flexors) | 2.33 ± 0.50 | 0.78 ± 0.67 | NR | 0.008 | ✓ | | | T1 = 4/52 |
| | | | Wrist Extension (Flexors) | 1.78 ± 0.44 | 0.33 ± 0.50 | NR | 0.009 | ✓ | | | |
| | | Chronic | Elbow Extension (Flexors) | 2.44 ± 0.88 | 2.11 ± 0.78 | NR | 0.083 | | ✓ | | |
| | | | Wrist Extension (Flexors) | 2.22 ± 1.09 | 1.56 ± 1.13 | NR | 0.063 | | ✓ | | |
| | MTS - R1 ° | Subacute | | - 52.78 ± 13.94 | - 15.56 ± 12.61 | NR | 0.008 | ✓ | | | |
| | MTS - R2 ° | | | - 6.67 ± 13.23 | - 0.56 ± 1.67 | NR | 0.109 | | ✓ | | |

Supplementary Table S4. Spasticity Outcomes from articles included in the analysis.

| | | | | | | | | | | | |
|---|---|---|---|---|---|---|---|---|---|---|---|
| | MTS – D | | Elbow Extension (Flexors) | 46.11 ± 20.88 | 15.00 ± 12.50 | NR | 0.015 | ✓ | | | |
| | MTS - R1 ° | | Wrist | 39.44 ± 11.02 | 61.67 ± 10.61 | NR | 0.007 | ✓ | | | |
| | MTS - R2 ° | | Extension | 57.22 ± 16.03 | 67.78 ± 6.67 | NR | 0.026 | ✓ | | | |
| | MTS – D | | (Flexors) | 17.78 ± 11.49 | 6.11 ± 6.97 | NR | 0.107 | | ✓ | | |
| | MTS - R1 ° | Chronic | Elbow | - 49.44 ±18.11 | - 24.44 ± 14.88 | NR | 0.007 | ✓ | | | |
| | MTS - R2 ° | | Extension | - 10.00 ± 8.29 | - 3.89 ± 6.01 | NR | 0.066 | | ✓ | | |
| | MTS – D | | (Flexors) | 39.44 ± 11.30 | 20.56 ± 13.33 | NR | 0.007 | ✓ | | | |
| | MTS - R1 ° | | Wrist | 26.11 ± 21.03 | 41.11 ± 20.88 | NR | 0.011 | ✓ | | | |
| | MTS - R2 ° | | Extension | 44.44 ± 20.07 | 51.11 ± 18.33 | NR | 0.059 | | ✓ | | |
| | MTS – D | | (Flexors) | 18.33 ± 8.29 | 10.00 ± 8.66 | NR | 0.070 | | ✓ | | |
| Rousseaux 2002 [82] | MAS (0-5) | Pre-Post | Wrist Flexors | 2.55 (0.83) | 1.63 (1.02) | 0.93 (0.57) | NR | | NR | | T1 = 2/52 |
| | | | | | 1.85 (0.90) | 0.70 (0.64) | NR | | NR | | T2 = 2/12 |
| | | | | | 2.20 (0.91) | 0.35 (0.46) | < 0.0001 | ✓ | | | T3 = 5/12 |
| Rousseaux 2005 [81] | MAS (0-5) | Pre-Post | Ankle Plantarflexors | 3.1 (0.89) | 2.37 (0.85) | 0.72 (0.50) | NR | ✓ | | | T1 = 2/52 |
| | | | | | 2.53 (0.91) | 0.56 (0.51) | NR | ✓ | | | T2 = 2/12 |
| | | | | | 2.78 (0.88) | 0.32 (0.45) | 0.0001 | ✓ | | | T3 = 5/12 |

*ƒ* - Calculated By Authors Based On Supplied Data, ^ - Data Supplied By Author Upon Request, D – the difference between R1 and R2 (Dynamic Range), NR – Not Reported Significance Result, NS - Not Significant, R1 - The Angle where the catch was beginning to be felt, R2 - the angle of maximum passive range of motion, s – Seconds, SD – Standard Deviation, SS - Statistically Significant.

Significance is reported as $p<0.05$ unless otherwise stated.

Supplementary Table S4. Spasticity Outcomes from articles included in the analysis.
