# Supplementary material for: The Effect of Botulinum Neurotoxin-A (BoNT-A) on Muscle Strength in Adult-Onset Neurological Conditions with Focal Muscle Spasticity: A Systematic Review
Source: Toxins (Basel). 2024 Aug 8;16(8):347. doi: 10.3390/toxins16080347 (PMC11359732; doi:10.3390/toxins16080347)
Supplement: Supplementary file 1 [file toxins-16-00347-s001.zip › Supplementary Table S5. Botulinum neurotoxin-A type, dose, dilution and Adjunctive therapies - Revised.pdf]

Supplementary Table S5: Botulinum neurotoxin-A type, dose, dilution, and adjunctive therapies (n = 54).

<table>
<tr><th>Author</th><th colspan="2">Treatment arm</th><th>BoNT-A: Type, Mean/Median dose (SD), (Range) [IQR], dilution, dose per muscle group</th><th>Adjunctive therapies or concurrent therapies</th></tr>
<tr><td rowspan="2">Baguley 2022 [63]</td><td>Experimental</td><td>UMNS post ABI</td><td rowspan="2">(Botox®) 198 U or (Dysport®) 738 U</td><td rowspan="2">N/A</td></tr>
<tr><td>Control</td><td>without ABI</td></tr>
<tr><td>Barden 2014 [64]</td><td colspan="2">Single arm</td><td>(Dysport®) 740 (500-1200) U; (Botox®) 200 (25–400) U</td><td>75% received therapy, 25% did not receive therapy.</td></tr>
<tr><td>Barden 2014 [65]</td><td colspan="2">Single arm</td><td>(Dysport®) 740 (500–1200) U; (Botox®) 200 (25–400) U</td><td>N/A</td></tr>
<tr><td rowspan="2">Bhakta 2000 [5]</td><td>Experimental</td><td>BoNT-A</td><td>(Dysport®) 1000 MU divided between EF, WF, FF</td><td rowspan="2">Possible existing PT treatments were unaltered.</td></tr>
<tr><td>Control</td><td>Placebo</td><td>Placebo, diluted in 10 mL 0.9% saline.</td></tr>
<tr><td rowspan="2">Baricich 2019 [45]</td><td>Group 1</td><td>ES of injected muscles + ES of TA</td><td rowspan="2">(Botox®) (50 - 120) U, 100 U diluted with 2 mL saline 0.9%
Individually tailored injection regimes.</td><td>ES (0.2 ms, 20 Hz) to the TA muscle (x1 PT session 60 mins/day) in first 1/52.</td></tr>
<tr><td>Group 2</td><td>ES of injected muscles</td><td>ES (0.2 ms, 4 Hz) on injected muscles immediately post injection.
x1 PT session 60 min/day, calf stretching, 20 min, gait training, aerobic + strengthening exercises.</td></tr>
<tr><td>Bernuz 2012 [19]</td><td colspan="2">Single arm</td><td>(Botox®) 200 U distributed in 2 points at each site.</td><td>N/A</td></tr>
<tr><td rowspan="2">Bollens 2013 [46]</td><td>Group 1</td><td>TNN</td><td>NA</td><td>PT remained unchanged.</td></tr>
<tr><td>Group 2</td><td>BoNT-A control group</td><td>100 UI/2 mL dilution
Sol 200 UI; TP 125 UI; FHL 75 UI</td><td>All participants but 2 (1 patient in each group) received regular PT.</td></tr>
<tr><td rowspan="2">Bumbea 2023 [66]</td><td>Experimental</td><td>BoNT-A + stretching + HEP</td><td>Incobotulinumtoxin, 200 U or Abobotulinumtoxin, max 1000 U (Incobotulinumtoxin 100 U equates to 400 U Abobotulinumtoxin). PQ - 200 U; FDP/FDS - 400 U; FCR - 150 U; D/pectoralis - 250 U, FPL - 100 U. Max 4 points injected. Doses were recommended and necessary according to the patient’s spasticity.</td><td>Group therapy for UL with spastic muscle x3 sessions, every 3/12.
Injections occurred in 3/12 intervals in 3 treatment stages.
Daily kinetic stretching therapy program for spastic muscles as HEP.</td></tr>
<tr><td>Control</td><td>ES + stretching + HEP</td><td>N/A</td><td>ES for antagonist muscles, x3 sessions, every 3/12.
Daily kinetic stretching therapy program for spastic muscles as HEP.</td></tr>
<tr><td rowspan="4">Carda 2011 [47]</td><td rowspan="2">Group 1</td><td rowspan="2">Casting (1/52) + BoNT-A</td><td rowspan="4">(Xeomin®) 100 (50- 140) IU, diluted 2 mL saline 0.9%
BB 250 U; BRA 200 U; FCR 150 U; Pt 150 U; FDP 200 U; FPL 50U.</td><td>All: 1/52 post injection, 30 mins of gait training + 20 mins PF muscle stretching, daily for 1/52 under guidance of senior PT</td></tr>
<tr><td>Casting: Below-knee fibreglass casts applied ankle in neutral supination–pronation and dorsiflexion.</td></tr>
<tr><td>Group 2</td><td>Taping (5 days) + BoNT-A</td><td>Taping: 5 days, adjusted daily by a trained PT</td></tr>
<tr><td>Group 3</td><td>Stretching + BoNT-A</td><td>Stretching: 30 mins x2/day for 1/52, guided by PT</td></tr>
<tr><td>Caty 2009 [67]</td><td colspan="2">Single arm</td><td>(Botox®) (400 – 1000 U), 500 U diluted in 1 mL saline
RF 200 U; HS 100 U; TS 200 U; FDL 100 U</td><td>PT remained unchanged throughout the study.</td></tr>
<tr><td>Chen 2020 [69]</td><td colspan="2">Single arm</td><td>Incobotulinumtoxin A or Onabotulinumtoxin A 100 U</td><td>N/A</td></tr>
</table>

Supplementary Table S5. Botulinum neurotoxin-A type, dose, dilution, and Adjunctive therapies (n = 54)

Chen 2022 [68]	Single arm		Incobotulinumtoxin A or Onabotulinumtoxin A 100 U	N/A
Cinone 2019 [32]	Experimental	Combined BoNT-A + isokinetic ankle DF training	Onabotulinumtoxin A<br>TS 107.14 (19.70) U; GN - Med 57.14 (18.29) U; GN- Lat 53.33 (14.71); Sol 47.5 (14.33) U	Isokinetic ankle DF training, 4/52, 5 days/week, total 20 sessions.
	Control	BoNT-A alone	Onabotulinumtoxin A<br>TS 120.70 (18.62) U; GN - Med 60.28 (16.50) U, GN- lat 52.24 (10.04) U; Sol - 53.50 (14.31) U	N/A
de Niet 2015 [70]	Single arm		(Dysport®) 500–750 MU, in 5 mL saline 0.9%.	Twice daily, 10 min calf stretching (with knees flexed and extended) for 18/52.
Diniz de Lima 2021 [48]	Experimental	BoNT-A then Saline + rehabilitation	(Prosigne®) 100 U 2 mL 0.9% sterile sodium chloride dilution, x4 intramuscular vials of 2 mL, 0.9% total of 400 IU<br>AM (100 U) TS 100 U; (¼ in head of each GN, 1/2 in Sol)	Individualised PT (i.e., strengthening/ stretching, proprioception, postural control, gait training, balance), stretching (Add, TS, QUADS, HS, GM) exercises once per day, x3 p/w sessions: x 3 sets 45 seconds of stretching + 15-sec interval training (Add, TS, QUAD, HS, GM).
	Control	Saline then BoNT-A + rehaibilitation	x4 intramuscular vials of 2 mL, 0.9% saline	
Fawzi 2023 [71]	Single arm		(Canitox®) 100 U diluted with 4 mL of saline.<br>Administered at baseline and 3-4/52.	NR
Franck 2021 [72]	Single arm		Abobotulinumtoxin A, 200 – 500 U<br>Administered once in 5/52 after usual therapy onset	BoNT-A and training 12/52 arm-hand regime based on level of impairment.
Gandolfi 2019 [49]	Experimental	Robot-assist UL training + BoNT-A	(Dysport®) 500 MU in 2 mL saline 0.9%	Robot-assisted UL Training: passive mobilisation + stretching UL (10min) + robot-assisted exercises (35 min) – EF/EE and reaching movement. Total x10 sessions (45 min/session, x2 p/w)
	Control	Conventional Rx + BoNT-A for 5/52 (45min x2 p/w)		UL passive mobilisation and stretching (10min) + UL exercises (35min) - single or multi-joint movements for scapula, shoulder, and elbow, performed in different positions (i.e., supine/ standing).<br>Total x10 sessions (45 min/session, x2 p/w)
Giray 2020 [44]	Experimental	BoNT-A + Lycra sleeve plus rehabilitation	Onabotulinumtoxin A<br>BB 60 IU; BRA 50 IU; PT 30 IU; FCR 30 IU; FCU 30 IU; FDS 30 IU; FDP 30 IU; FPL 20 IU; PQ 20 IU<br>Injected under ultrasound and ES-guidance.	Lycra sleeve: 8 hr./day, 5 days p/w, 3/52.<br>Therapy: passive, active and active-assistive range-of-motion, stretching exercises for shoulder, elbow, wrist, facilitation, and inhibition techniques, neuromuscular ES, strengthening for affected UL + OT 2 hrs./day 5 days p/w.
	Control	BoNT-A + Only rehabilitation control group		Therapy: passive, active and active-assistive range-of-motion, stretching exercises for shoulder, elbow, wrist, facilitation, and inhibition techniques, neuromuscular ES, strengthening for affected UL + OT 2 hrs./day 5 days p/w.
Gracies 2009 [50]	Group 1		Onabotulinumtoxin A, 100 MU/mL dilution,<br>0.4cc/site, 4-quadrant injection	Established PT/OT remained unchanged.
	Group 2		Abobotulinumtoxin A, 100 MU/mL dilution,<br>0.4cc/site, 4 sites endplate band	
	Group 3		Incobotulinum toxin A, 20 MU/mL dilution,<br>2cc/site, 4-quadrant injection	
Hameau 2014 [33]	Single arm		Onabotulinumtoxin A (Botox®), Dose - NR.	N/A

Supplementary Table S5. Botulinum neurotoxin-A type, dose, dilution, and Adjunctive therapies (n = 54)

Intisio 2014 [73]	Single arm		Incobotulinumtoxin A (Xeomin®) 840 IU (770-840 IU), dilution 2mL of 0.9% saline, 1mL contained 50 IU.<br>BB, BRA, BR (50-150 IU); PT (40–70 IU); FCU/FCR (50-80 IU); FDS/FDP/FPL/FPB (50-100 IU); GN – Med /GN - Lat, Sol (50-100 IU); TP (30–100 IU); ADLBM (100-120 IU); RF (40-50 IU); BF (50-100 IU); FDL/ FHL (30-50 IU).	Usual PT, 3x/week.
Kaji 2022 [51]	Group 1		A2NTX prepared from subtype A2, 50 U (diluted in 6 mL of saline). TP, GN-Med – 150 U. Injected with use of an EMG device.	Regular rehabilitation therapy continued
	Group 2		Onabotulinumtoxin A (Botox®) derived from A1, 50 U (diluted in 6 mL of saline). TP, GN-Med – 150 U.<br>Injected with use of an EMG device.	
Kulkarni 2004 [74]	Single arm		(80-400 U), 2mL per vial of 100 U, 1 mL syringe in 50 ml	PT 30 mins, x2 daily - inpatient admission. PT x2 weekly in the community.
Lannin 2020 [53]	Experimental	BoNT-A + casting + movement training	(Botox®) max 600 U, 100 U (max volume per site = 0.5-1.0 mL). Muscles crossing the wrist.	BoNT-A + 2/52 serial casting applied to place the wrist in maximal extension for 2/52, + 10/52 of movement training (ES and progressive resistance training). 60 mins per day, 7 days p/w, 10/52 (~70 hrs. total).
	Control	BoNT-A + HEP		Usual care (not stipulated)
Lannin 2022 [52]	Experimental	BoNT-A + 2/52 of serial casting + 10/52 movement training	(Botox®) max 600 U, 100 U (max volume per site = 0.5-1.0 mL). Muscles crossing the wrist.<br>FCR E: 49(19), C: 47(16); FCU E: 48(19) C: 50(18); FDS 52(18) C: 53(21) FDP E: 47(15) C: 51 (20); FPL E: 32(21) C: 38 (22); ECRL E: 34(22) C: 20(14) (supplied as supplementary material)	BoNT-A + 2/52 serial casting followed + 10/52 of movement training. 2/52 serial casting in max wrist extension + 10/52 of movement training. 60 mins per day, 7 days p/w, for 10/52 (~70 hrs. total).
	Control	BoNT-A + HEP		HEP + x1 follow-up phone call to encourage independence. HEP (7 stretches, 8 arm, hand exercises). Participants did not receive other UL rehabilitation.
Lee 2018 [75]	Single arm	BoNT-A + ES	Onabotulinumtoxin A (Botox®), max 360 U, 100 U diluted 2 mL saline (5 U/0.1 mL). Individual patient injection dosages reported in Table 3. Muscles selected according to individual's spasticity. ≥ 1 elbow flexor muscles (BB, BR, BRA).	2/52 post BoNT-A to finger +/- wrist flexors, ES of FE + wrist brace for 4/52 (5 days p/w; 30 mins).
Leung 2019 [54]	Experimental	BoNT-A + Serial casting + motor training	(Botox®) (60-100U) 1 60 Sol 100U; TP 100 U<br>Muscles selected according to the spasticity of each patient.	BoNT-A + serial casting + splinting and motor training.<br>Serial casting 5 days post injection, cast changed every 7 days.<br>Post casting a splint was worn 24/7 (removed for therapy/skincare)<br>Motor training: individualised PT +/- ankle strength, standing, walking.
	Control	BoNT- A waitlist 6/52 + serial casting + motor training		After 6/52 wait - BoNT-A + serial casting, + splinting and motor training.<br>Motor training: individualised PT +/- ankle strength, standing, walking.
Lim 2016 [90]	Subacute Group		(Botox®) 200 U, 100 U vial diluted 2 mL normal saline.<br>Injected muscles and appropriate doses chosen for the individual muscle. ≥ 1 EF muscles (BB, BR, BRA) + ≥ 1 WF muscles (FCR, FCU) or ≥ 1 FF muscles (FDP, FDS, FPL).	Inpatient or outpatient rehabilitation treatment 2 days p/w
	Chronic group			

Supplementary Table S5. Botulinum neurotoxin-A type, dose, dilution, and Adjunctive therapies (n = 54)

López de Munain 2019 [76]	Single arm		Abobotulinumtoxin A (92.0%) Ona botulinum toxin A (6.0%), incobotulinumtoxinA (2%). 250 U (100; 1,000); 50 U (50 -250) and 100 U (100-100) respectively. Investigators choice of BoNT-A product, dose, volume, number of injections. Median number of injections = 3.		NR
Macher 2021 [55]	Experimental	Exercise group	Incobotulinumtoxin 230 MU (120 MU – 900 MU)<br>80 MU diluted with 2 mL of saline solution.		Incobotulinum toxin + EF exercise. EF – 1 min before and 1min after injection. EF exercise – passive/active movement +/- weight or repetitive lifting of 0.5L plastic water bottle.
	Control	Non-exercise group			Incobotulinum toxin only. 7 patients (3 patients in the exercise group) received PT at varying intervals and extent during the observation period.
Marque 2019 [77]	Single arm		Abobotulinumtoxin A 875 (318) U max 1000 U<br>Onabotulinumtoxin A 214 (92) U max 400 U		92% of participants received regular PT
Mancini 2005 [56]	Group 1	Low Dose	166.7 (30.9) U	GN-Med, GN-Lat 50 U; Sol 50 U; TP 50 U; TA 50 U; FDL/FDB 50 U; FHL 25 U; EH 25 U	Ceased 2 months prior to the study and remained no therapy throughout study
	Group 2	Medium Dose	321.7 (92) U	GN-Med, GN-Lat 100 U; Sol 75 U; TP 100 U; TA 100 U; FDL/FDB 75 U; FHL 50 U; EH 50 U	
	Group 3	High Dose	540 (124.2) U	GN-Med, GN-Lat 200 U; Sol 100 U;<br>TP 200 U; TA 150 U; FDL/FDB 100 U; FHL 75 U; EH 74 U	
Meythaler 2009 [57]	Experimental	BoNT-A + therapy for first 12/52 then Placebo + therapy	(Botox®) 300 U – 400 U, 100 U in 1-mL syringes unlabelled.		BoNT-A + OT 1 hr. x2 weekly, 12/52<br>Splints used for participants unable to extend wrist >15° 4-hrs-on (1-2 hrs. off) Phase 2: the splint-wearing schedule was again 4 hrs. on and 1 - 2 hrs. off with full-time night wear.
	Control	Placebo + therapy in the first 12/52 then BoNT-A + therapy	Placebo 1-mL syringes unlabelled preservative-free saline.		Placebo injection + therapy 1 hr. x2 weekly, 12/52<br>Splints used for participants unable to extend wrist >15° 4-hrs-on (1-2 hrs. off) Phase 2: the splint-wearing schedule was again 4 hrs. on and 1 - 2 hrs. off with full-time night wear.
Miscio 2004 [78]	Single arm		(Botox®)100-190 MU, dilution of 100 MU in 2.5ml of saline<br>(Dysport®)100-500 MU, dilution of 500 MU in 2.5ml of saline. (1 MU - Botox® equivalent to 3 MU - Dysport®)		All patients received intensive PT of injected UL
Pandyan 2002 [30]	Single arm		(Botox®) 100-190 MU, dilution of 100 MU in 2.5ml. (BB 70 U); BR 56.5 U; EDL 83.3 U.<br>(1 MU of Botox® equivalent to 3 MU of (Dysport®)<br>(Dysport®) dilution 500 MU in 2.5 mL of saline 100-500 MU		N/A

Supplementary Table S5. Botulinum neurotoxin-A type, dose, dilution, and Adjunctive therapies (n = 54)

Paolucci 2021 [79]	Group 1	Robot	(Botox®/ Dysport®) Dose NR, diluted in 2CC of saline. Number of injection sites and dose were determined at the discretion of the investigator, physician.	Robotics (end effector system + exoskeleton) + conventional outpatient rehabilitation therapy. 30 mins, 3 days p/w, total of 20 sessions.
	Group 2	Robot + BoNT-A		BoNT-A + robotic treatment + conventional therapy 30 min, 3 days p/w, total of 20 sessions.
Picelli 2021 [80]	Single Arm		(Botox®/ Dysport®) Dose NR, dilution 2 mL per vial (67.5%)	N/A
Reiter 1996 [91]	Single Arm		(Botox®) 165 MU (100 - 210 MU) diluted saline of 5 MU. Under EMG guidance. 3-5 UL flexor muscles.	NR
Rousseaux 2002 [82]	Single Arm		(Botox®) 200 - 300 U, diluted 100 U/ml.	PT/OT kept constant throughout study (5/12).
Rousseaux 2005 [81]	Single Arm		(Botox®) 300 U; Sol 123.8 U; GN 78.9 U; TP 47.8 U; FDL 52.7 U; FHL 52.7 U; TA 54.3 U.	PT was kept as constant throughout the study, 5/12
Rousseaux 2007 [83]	Single Arm		(Botox®) 400 U, 50 U/ml. Sol + GN 160 U, TP 40 U, FDL 40 U, AL/AM 80-100 U. Individually tailored injection regimes.	PT was kept as constant throughout the study, 5/12.
Sarzynska-Dlugosz 2020 [84]	Single arm		Abobotulinumtoxin A (Dysport®) 2307.8 (798.0) U.<br>Inj. 1 - 853.4 U (231.4); Inj. 2 - 838.9 U (248.4), Inj. 3 - 813.8 U (264.1)	Investigators could alter or initiate PT at any time according to clinical need.
Servelhere 2018 [85]	Group 1		(Dysport®) 1,110.0 (435.1) U; GN 341 (98.9) U; Add 488.4 (165.7) U; Sol 145.4 (35.8) U; HS 402.5 (146.7) U; TP 130 (64.1) U; QUADS 356 (126.8) U; EHL 106.6 (23) U; FDL 130 (14.1) U; FDB 120 (0) U; FHL 80 U; QL 200 U; TA 220 U; FHB 80 U; QL 200 U; TA 220 U.<br>Administered using palpatory guidance.	NR
	Group 2		(Dysport®) 345.4 (131.2) U; GN 200 (44.7 U); Add 181.2 (59.3 U); Sol 50 U; TP 100 U.<br>Administered using palpatory guidance.	
Shaw 2010 [58]	Experimental	BoNT-A UL therapy (1 hr. x2 p/w, 4/52)	(Dysport®) max 500 U, 100 U or 200 U/mL<br>Repeat injections if necessary, after reassessment if clinically indicated until 12/12 follow up.<br>Initial treatment 200 U [100 – 300]; 3 months 300 U [150 -400]; 6 months: 300 U [150 – 450]; 9 months: 300 U [188 - 450]	Therapy by trained study therapists 1 hr. per day, x2, 4/52.<br>Program 1: For participants with no active function at baseline (ARAT 0 - 3) and consisted of stretching (20 mins), positioning (10 mins), and passive/active, assisted UL activity (20 mins).<br>Program 2: For participants with function at baseline (ARAT 3) stretching (10 mins) + task-oriented practice (40 mins) + HEP on non-therapy days.
	Control	Therapy Alone	NA	4/52 UL therapy (1 hr. x2 per week)
Shaw 2011 [59]	Experimental	BoNT-A + 4/52 UL therapy (1 hr. x2 p/w)	(Dysport®) max 500 U, 100 U or 200 U/mL<br>Repeat injections, if necessary, after reassessment if clinically indicated. Initial treatment 200 U [100 – 300]; 3 months 300 U [150 -400]; 6 months: 300 U [150 – 450]; 9 months: 300 U [188 - 450]	4/52 standardised evidence-based UL therapy program<br>(1 hr. x2 per week)
	Control	Therapy Alone	NA	4/52 UL therapy alone (1 hr. x2 p/w)
Simpson 1996 [62]	Experimental	BoNT	(Botox®) 75, 150, or 300 U into BB, FCR, FCU	Stable clinical course for 2/12 before study + maintained spasticity treatments (PT) throughout study.
	Control	Placebo	Placebo into BB, FCR, FCU	
Slawek 2005 [86]	Single arm		(Botox®) 255 (185–300) U. Administered according to individual spasticity pattern	Daily PT unchanged.

Supplementary Table S5. Botulinum neurotoxin-A type, dose, dilution, and Adjunctive therapies (n = 54)

Tsuchiya 2016 [87]	Single arm		(Botox®) max 240 U, dilution 100 U with 4 mL of saline (2.5 U/0.1 mL). Administered according to individuals' spasticity under EMG guidance.	Pre- injection: Task-orientated therapy<br>Post-injection: Task-orientated therapy + EMG-FES for unilateral paretic UL function, 40 - 60 mins, 1-2x weekly, 4/12.
Turcu-Stiolica 2021 [60]	Experimental	BoNT- A + PT	Incobotulinumtoxin A (Xeomin ®) 200 U	Specific PT program (i.e., specific stretching exercises), adjusted PT program at 3/12
	Control	Baclofen+ conventional therapy	Baclofen (10 mg - 60 mg daily)	Conventional therapy (unspecified)
Wallace 2020 [61]	Experimental	BoNT-A + UL standardised PT 4/52	Onabotulinumtoxin A (Botox ®) 115 (40-190) U, 100 U diluted 2 mL saline.<br>Median of 5 muscles injected (Range:1-8).	PT (10 sessions, 45 mins – 1.5 hrs.) intensive, standardised PT over 4/52.<br>PT: Strength/ functional training, 3x10 reps WE, FE, grip 60% - 80% of isometric MVC, tasks relevant to individual goals.
	Control	PT only + placebo	Placebo: 151 (55-290) U, Median muscles injected 7 (4-12)	PT as above
Wang 2002 [88]	Single arm		(Botox®) 140 (80–200) U dilution of 100 U in 2 mL saline (spread between elbow, wrist, finger flexors)	Pre-injection rehabilitation schedules were continued in each patient.
Woldag 2003 [89]	Single arm		(Dysport®) 480 MU (all patients). 2.5 ml saline vials of 200 MU/ml. 120 MU into FCR, FCU, FDP, FD.	All patients received OT/ PT two to three times p/w
Yan 2018 [4]	Group 1	Baclofen + PT	500 U<br>EMG used to identify each muscle.	PT: locomotor training + intensive task-specific training or rehabilitation supervised by PTs, 6/52
	Group 2	BoNT-A + PT		
	Group 3	PT alone		

ABI – Acquired Brain Injury; Add – Adductors; ADLBM – Adductor Digitorum Longus Brevis Magnus; AL – Adductor Longus; AM – Adductor Magnus; ARAT - Action Research Arm Test; BB - Biceps Brachii; BF – Biceps Femoris; BI – Brain injury; BoNT- A- Botulinum-toxin A;BR – Brachioradialis; BRA – Brachialis; C – Control group; D – Deltoid; DF – Dorsiflexion; EDL – Extensor Digitorum Longus; EE – Elbow Extensors; EF – Elbow Flexors; EH – Extensor Hallucis; EHL – Extensor Hallucis Longus; EMG – Electromyography; EMG-FES - Electromyography–Functional Electrical Stimulation Interface; ES – E-Stims; FCR – Flexor Carpi Radialis; FCU – Flexor Carpi Ulnaris; FD – Flexor Digitorum; FDB - Flexors Digitorum Brevis; FDL – Flexor Digitorum Longus; FDP – Flexor Digitorum Profundus; FDS – Flexor Digitorum Superficialis; FE – Finger Extension; FES – Functional electrical stimulation; FF – Finger flexion; FHB – Flexor Hallucis Brevis; FHL – Flexor Hallucis Longus; FPB – Flexor Pollicis Brevis; FPL – Flexor Pollicis Longus; GM – Gluteus Maximus; GN – Gastrocnemius; HEP – Home exercise program; HS – Hamstrings; Hz – Hertz; inj – injection; IU – international unit; lat – lateral; LL – Lower limb; med – medial; Mg - milligrams; Mins – Minutes; mL - milliliters; MU - Mouse units; MVC - Maximal Voluntary Contraction; N/A – Not Applicable; NR – Not Reported; OT – Occupational Therapy/Occupational Therapist; p/w – per week; PL – Palmaris Longus; PL – Pollicis Longus; PQ – Pronator Quadratus; PT – Physiotherapy/Physiotherapist; Pt – Pronator Teres; PT- Physiotherapy/Physiotherapist; QL - Quadratus Lumborum; QUADS – Quadriceps; RF – Rectus Femoris; Rx – Treatment; SD – Standard deviation; Sol – Soleus; TA – Tibialis Anterior; TF - Thumb Flexors; TNN – Tibial nerve neurotomy; TP – Tibialis Posterior; TS – Triceps Surae; U – units; UL – Upper Limb; UMNS – Upper Motor Neuron Syndrome; WE – Wrist Extension; WF – Wrist Flexion.

Supplementary Table S5. Botulinum neurotoxin-A type, dose, dilution, and Adjunctive therapies (n = 54)
