# Supplementary material for: The Effect of Botulinum Neurotoxin-A (BoNT-A) on Muscle Strength in Adult-Onset Neurological Conditions with Focal Muscle Spasticity: A Systematic Review
Source: Toxins (Basel). 2024 Aug 8;16(8):347. doi: 10.3390/toxins16080347 (PMC11359732; doi:10.3390/toxins16080347)
Supplement: Supplementary file 1 [file toxins-16-00347-s001.zip › Supplementary Table S6. Search Strategy All Databases March 2024 - Revised.pdf]

Supplementary Table S6. Search Strategy All Databases March 2024

All searches were limited to humans and English.

CINAHL <1990 - March 19 2024>

S1	(MH "Nervous System Diseases+") OR (MH "Diagnosis, Neurologic+") OR neurological disorder OR TI neurological disorder OR AB neurological disorder OR neurological disease OR TI neurological disease OR AB neurological disease
S2	MH Stroke OR TI Stroke OR AB Stroke OR lacunar stroke OR subarachnoid haemorrhage OR subdural haemorrhage OR subdural haematoma OR cerebrovascular disorders* OR haemorrhage OR intracranial haemorrhage OR intracranial thrombosis OR intracranial embolism OR brain hypoxia OR brain ischemia OR cerebral haemorrhage OR intracranial embolism OR intracranial thrombosis OR intracranial arterial diseases OR cerebral arterial diseases OR carotid artery diseases OR basal ganglia cerebrovascular disease OR cerebral ischemia OR cerebral ischemia OR intracranial sinus thrombosis OR cerebrovascular accident OR CVA OR cerebral infarct* OR brain infarct* OR intracranial haemorr* OR intra-cranial haemorr* OR intracranial hemorr* OR intra-cranial hemorr* OR cerebral haemorr* OR cortical haemorr* OR cortical ischaem* OR cerebral ischaem* OR cerebral ischem* OR brain ischaem* OR cerebrovasc* disord* OR cerebral aneurysm* OR brain aneurysm* OR hypoxic brain injur* OR anoxic brain injur* OR subdural haem* OR subdural Hem* OR intracranial thromb* OR intra-cranial thomb* OR intra-cranial emb* OR intracranial emb* OR brain lesion* OR cerebral lesion* OR cortical lesion* OR cerebrovascular disease OR basal ganglia cerebrovascular disease OR basal ganglia h?morrhage OR brain ischemia OR brain infarction OR brain stem infarctions OR lateral medullary syndrome OR cerebral infarction OR anterior cerebral artery infarction OR middle cerebral artery infarction OR posterior cerebral artery infarction OR brain hypoxia-ischemia OR carotid artery diseases OR carotid artery thrombosis OR arteriovenous malformations OR intracranial arteriovenous malformations OR putaminal hemorrhage OR putaminal haemorrhage OR poststroke OR poststroke OR apoplex* OR cerebral vasc* OR cerebralvasc* OR cva OR SAH OR cerebr* OR cerebell* OR vertebrobasilar artery OR intracerebral OR intracran* OR intra-cranial OR basal gangli* OR hemisphere* OR subarachnoid
S3	MH multiple sclerosis OR TI multiple sclerosis OR AB multiple sclerosis
S4	MH cerebral palsy OR TI cerebral palsy OR AB cerebral palsy
S5	(MH "Hemiplegia") OR (MH "Paralysis+") OR "hemiparesis" OR TI hemipare* OR AB hemipare* OR TI acquired brain injur* OR AB acquired brain injur* OR TI traumatic brain injur* OR AB traumatic brain injur* OR brain injur*OR head injur* OR acquired brain injur* OR brain damage OR brain stem injur* OR cerebell* injury OR diffuse axonal injur* OR brain haemorrhage OR brain hypoxia OR brain cortex lesion OR brain cyst OR brain damage OR brain cancer OR brain tumour OR cerebrovascular trauma OR “cerebral trauma” OR cerebral damage
S6	(MH "Brain Injuries+") OR "acquired brain injury" OR AB Brain injur* OR brain injur* OR head injur* OR AB acquired brain injur* OR TI traumatic brain injur* OR AB traumatic brain injur* OR brain damage OR brain stem injur* OR cerebell* injury OR diffuse axonal injur* OR brain haemorrhage OR brain hypoxia OR brain cortex lesion OR brain cyst OR brain damage OR brain cancer OR brain tumour OR cerebrovascular trauma OR “cerebral trauma” OR cerebral damage OR TBI OR ABI OR Acquired brain injur* OR traumatic brain injur*
S7	TI acquired brain injur* OR AB acquired brain injur* OR TI traumatic brain injur* OR AB traumatic brain injur* OR brain injur*OR head injur* OR acquired brain injur* OR brain damage OR brain stem injur* OR cerebell* injury OR diffuse axonal injur* OR brain haemorrhage OR brain hypoxia OR brain cortex lesion OR brain cyst OR brain damage OR brain cancer OR brain tumour OR cerebrovascular trauma OR “cerebral trauma” OR cerebral damage OR spinal cord injury OR spinal cord degenerationS2
S8	(MH "Muscle Spasticity") OR "spasticity" OR AB spasticity OR TI spasticity OR TI muscle spasticity OR AB muscle spasticity OR muscle hypertonia OR muscle rigidity OR muscle tonus OR spasm OR dystonia OR paraparesis OR spastic* OR hypertonicity OR muscle overactivity OR upper motor neuron* syndrome OR stretch reflex OR hypertonia OR muscle tone OR muscle spasm OR paraplegia OR hyperton* OR spastic paresis OR spasticity OR myotatic reflex OR stretch reflex OR (MH muscle spasm) OR TI muscle spasm OR AB muscle spasm OR MH hyperton* OR TI hyperton* OR AB hyperton* OR MW muscle rigidity OR TI muscle rigidity OR AB muscle rigidity OR MW muscle tone OR TI muscle tone OR AB muscle tone OR MW dystonia OR TI dystonia OR AB dystonia OR (MH "Reflex, Stretch") OR "myotactic reflex"
S9	(MH "Muscle Strength+") OR (MH "Grip Strength") OR (MH "Muscle Weakness+") OR (MH "Muscle, Skeletal+") OR "strength" OR power OR AB power OR "muscle power" OR TI muscle weakness OR TI muscle, skeletal OR AB

	muscle weakness OR AB muscle, skeletal OR muscle contraction OR eccentric contraction OR concentric contraction OR isometric contraction OR isotonic contraction OR isokinetic contraction OR muscle contraction OR Muscle weakness OR weakness OR paraparesis OR paresis OR strength OR muscle strength OR hand strength OR pinch strength OR motor control OR strength OR motricity index OR dynamo* OR Manual muscle test OR Musc* power OR force generation OR torque OR force production OR force OR (muscle* adj4 (contraction* or voluntary activation or force* or power)) OR maximal voluntary contraction* OR (MH "Muscle Contraction+") OR (MH "Eccentric Contraction") OR (MH "Concentric Contraction") OR (MH "Isotonic Contraction+") OR (MH "Isometric Contraction")
S10	(MH "Botulinum Toxins") OR botulinum OR botulinum toxin OR BTX-A OR BTXA OR BoNT-A OR BONTA OR dysport OR Xeomin OR AbobotulinumtoxinA OR OnabotulinumtoxinA OR IncobotulinumtoxinA
S11	S1 OR S2 OR S3 OR S4 OR S5 OR S6 OR S7
S12	S5 OR S9
S13	S8 AND S10 AND S11 AND S12

Embase <1974 to 2024 March 19>

1	neurological disorder.mp. or exp neurologic disease/
2	neurological disorder.mp. or exp neurologic disease/
3	multiple sclerosis.mp. or exp multiple sclerosis/
4	cerebral palsy.mp. or exp cerebral palsy/
5	hemiparesis.mp. or exp hemiparesis/ or exp hemiplegia/ or paresis.mp.
6	Hemiparesis/ or hemiplegia/ or hemipleg*/ or hemipar*/ or paralysis/ or paraparesis/ or mono paresis/ or exp muscle weakness/ or exp limb weakness/ or exp arm weakness/ or exp weakness/ or exp leg weakness/ or atrophy/ or paraplegia/ or paresis/ or spastic paraplegia/ or spastic paresis/ or paralysis/
7	Stroke/ or stroke, lacunar/ or subarachnoid h*morrhage/ or subarachnoid h*morrhage/ or subdural h*morrhage/ or subdural h*matoma/ or cerebrovascular disorders*/ or h*morrhage/ or intracranial h*morrhage/ or intracranial thrombosis/ or intracranial embolism/ or brain hypoxia/ or brain isch*mia/ or cerebral h*morrhage/ or intracranial embolism/ or intracranial thrombosis/ or intracranial arterial diseases/ or cerebral arterial diseases/ or carotid artery diseases/ or basal ganglia cerebrovascular disease/ or cerebral isch*mia/ or intracranial sinus thrombosis/ or cerebrovascular accident/ or CVA/ or cerebral infarct*/ or brain infarct*/ or intracranial h*morr*/ or intra-cranial h*morr*/ or intra-cranial h*morr*/ or cerebral haemorr*/ or cortical haemorr*/ or cortical ischaem*/ or cerebral ischaem*/ or cerebral ischem*/ or brain ischaem*/ or cerebrovasc* disord*/ or cerebral aneurysm*/ or brain aneurysm*/ or hypoxic brain injur*/ or anoxic brain injur*/ or subdural haem*/ or subdural H*m*/ or intracranial thromb*/ or intra-cranial thomb*/ or intra-cranial emb*/ or intracranial emb*/ or brain lesion*/ or cerebral lesion*/ or cortical lesion*/ or cerebrovascular disease/ or basal ganglia h*morrhage/ or brain isch*mia/ or brain infarction/ or brain stem infarctions/ or lateral medullary syndrome/ or infarction, anterior cerebral artery/ or infarction, middle cerebral artery/ or infarction, posterior cerebral artery/ or hypoxia-ischemia, brain/ or carotid artery diseases/ or carotid artery thrombosis/ or arteriovenous malformations/ or intracranial arteriovenous malformations/ or putaminal h*morrhage/ or putaminal h*morrhage/ or poststroke/ or post-stroke/ or apoplex*/ or cerebral vasc*/ or cerebralvasc*/ or cva/ or SAH/ or cerebr*/ or cerebell*/ or vertebrobasilar artery/ or intracerebral/ or intracran*/ or intra-cranial/ or basal gangli*/ or hemisphere*/ or subarachnoid/ or arteriovenous/ or arterio-venous/ or brain tumour/ or brain tumor/
8	exp traumatic brain injury/ or exp brain injury/ or exp brain damage/ or TBI/ or ABI/ or Acquired brain injur*/ or traumatic brain injur*/ or head injur*/ or acquired brain injur*/ or brain damage/ or brain stem injur*/ or cerebell* injury/ or diffuse axonal injur*/ or brain haemorrhage/ or brain hypoxia/ or brain cortex lesion/ or brain cyst/ or brain damage/ or brain cancer/ or brain tumour/ or cerebrovascular trauma/ or cerebral trauma/ or cerebral damage.mp. [mp=title, abstract, heading word, drug trade name, original title, device manufacturer, drug manufacturer, device trade name, keyword heading word, floating subheading word, candidate term word]
9	exp cerebrovascular accident/ or exp cerebrovascular disease/ or exp cerebrovascular disorders/ or exp brain ischemia/ or cerebrovasc.mp. or exp infarction/ or exp cerebrovascular accident/
10	exp muscle, skeletal/ or exp grip strength/ or exp pinch strength/ or exp muscle strength/ or exp hand strength/ or strength.mp. or exp strength/
11	muscle contraction/ or eccentric contraction/ or concentric contraction/ or isometric contraction/ or isotonic contraction/ or isokinetic contraction/ or muscle contraction/ or Muscle weakness/ or weakness/ or paraparesis/ or paresis/ or strength/ or muscle strength/ or hand strength/ or pinch strength/ or motor control/ or torque/ or strength/ or motricity index/ or dynamo*/ or Manual muscle test/ or Musc* power/ or force generation/ or force production/ or force.mp. or (muscle* adj4 (contraction* or voluntary activation or force* or power)).mp. or maximal voluntary contraction*.mp. [mp=title, abstract, heading word, drug trade name, original title, device manufacturer, drug manufacturer, device trade name, keyword heading word, floating subheading word, candidate term word]
12	spasticity.mp. or exp spasticity/ or exp spastic paresis/ or exp muscle hypertonia/ or exp muscle rigidity/
13	muscle spasticity/ or muscle hypertonia/ or muscle rigidity/ or muscle tonus/ or spasm/ or dystonia/ or paraparesis/ or spastic*/ or hypertonicity/ or muscle overactivity/ or upper motor neuron* syndrome/ or stretch reflex/ or hypertonia/ or muscle tone/ or muscle spasm/ or paraplegia/ or hyperton*/ or spastic paresis/ or spasticity/ or myotatic reflex/ or stretch reflex/

14	botulinum toxin.mp. or exp botulinum toxin/ or exp botulinum toxin A/
15	Botulinum/ or botulinum toxin/ or BTX-A/ or BTXA/ or BoNT-A/ or BONTA/ or disport/ or Xeomin/ or AbobotulinumtoxinA/ or OnabotulinumtoxinA/ or IncobotulinumtoxinA/
16	2 or 3 or 4 or 5 or 6 or 7 or 8 or 9
17	5 or 6 or 10 or 11
18	12 or 13
19	14 or 15
20	16 and 17 and 18 and 19

Cochrane Database < 1996 - March 19th 2024>

1	MeSH descriptor: [Muscle Spasticity] explode all trees
2	muscle spasticity OR muscle hypertonia OR muscle rigidity OR muscle tonus OR spasm OR dystonia OR paraparesis OR spastic* OR hypertonicity OR muscle overactivity OR upper motor neuron* syndrome OR stretch reflex OR hypertonia OR muscle tone OR muscle spasm OR paraplegia OR hyperton* OR spastic paresis OR spasticity OR myotatic reflex OR stretch reflex
3	MeSH descriptor: [Muscle Strength] explode all trees
4	muscle contraction OR eccentric contraction OR concentric contraction OR isometric contraction OR isotonic contraction OR isokinetic contraction OR muscle contraction OR Muscle weakness OR weakness OR paraparesis OR paresis OR strength OR muscle strength OR hand strength OR pinch strength OR motor control OR strength OR motricity index OR dynamo* OR Manual muscle test OR MMT OR "Manual-muscle-test" OR Musc* power OR force generation OR force production OR force OR (muscle* adj4 (contraction* or voluntary activation or force* or power)) OR maximal voluntary contraction* OR isokinetic OR torque OR anatagonist OR agonist
5	MeSH descriptor: [Nervous System Diseases] explode all trees
6	neurological disorder OR neurological disorder*
7	incomplete spinal cord injury OR SCI OR spinal degener* OR lower motor neurone
8	MeSH descriptor: [Stroke] explode all trees
9	Stroke OR lacunar stroke OR subarachnoid haemorrhage OR subdural haemorrhage OR subdural haematoma OR cerebrovascular disorders* OR haemorrhage OR intracranial haemorrhage OR intracranial thrombosis OR intracranial embolism OR brain hypoxia OR brain ischemia OR cerebral haemorrhage OR intracranial embolism OR intracranial thrombosis OR intracranial arterial diseases OR cerebral arterial diseases OR carotid artery diseases OR basal ganglia cerebrovascular disease OR cerebral ischemia OR cerebral ischemia OR intracranial sinus thrombosis OR cerebrovascular NEXT accident* OR CVA OR cerebral infarct* OR brain infarct* OR intracranial haemorr* OR intra-cranial haemorr* OR intracranial hemorr* OR intra-cranial hemorr* OR cerebral haemorr* OR cortical haemorr* OR cortical ischaem* OR cerebral ischaem* OR cerebral ischem* OR brain ischaem* OR cerebrovasc* disord* OR cerebral aneurysm* OR brain aneurysm* OR hypoxic brain injur* OR anoxic brain injur* OR subdural haem* OR subdural Hem* OR intracranial thromb* OR intra-cranial thomb* OR intra-cranial emb* OR intracranial emb* OR brain lesion* OR cerebral lesion* OR cortical lesion* OR cerebrovascular disease OR basal ganglia cerebrovascular disease OR basal ganglia h?morrhage OR brain ischemia OR brain infarction OR brain stem infarctions OR lateral medullary syndrome OR cerebral infarction OR anterior cerebral artery infarction OR middle cerebral artery infarction OR posterior cerebral artery infarction OR brain hypoxia-ischemia OR carotid artery diseases OR carotid artery thrombosis OR arteriovenous malformations OR intracranial arteriovenous malformations OR putaminal hemorrhage OR putaminal haemorrhage OR poststroke OR post-stroke OR apoplex* OR cerebral vasc* OR cerebralvasc* OR cva OR SAH OR cerebr* OR cerebell* OR vertebrobasilar artery OR intracerebral OR intracran* OR intra-cranial OR basal gangli* OR hemisphere* OR subarachnoid OR arteriovenous OR arterio-venous OR brain tumour OR brain tumor
10	MeSH descriptor: [Brain Injuries, Traumatic] explode all trees
11	TBI OR ABI OR Acquired brain injur* OR traumatic brain injur*
12	brain injur*OR head injur* OR acquired brain injur* OR brain damage OR brain stem injur* OR cerebell* injury OR diffuse axonal injur* OR brain haemorrhage OR brain hypoxia OR brain cortex lesion OR brain cyst OR brain damage OR brain cancer OR brain tumour OR cerebrovascular trauma OR “cerebral trauma” OR cerebral damage
13	MeSH descriptor: [Multiple Sclerosis] explode all trees
14	multiple sclerosis
15	MeSH descriptor: [Cerebral Palsy] explode all trees
16	cerebral palsy
17	MeSH descriptor: [Botulinum Toxins] explode all trees
18	botulinum OR botulinum toxin OR BTX-A OR BTXA OR BoNT-A OR BONTA OR dysport OR Xeomin OR AbobotulinumtoxinA OR OnabotulinumtoxinA OR IncobotulinumtoxinA
19	MeSH descriptor: [Paresis] explode all trees
20	hemiparesis OR hemiplegia OR hemipleg* OR hemipar* OR paralysis OR paraparesis OR mono paresis OR muscle weakness OR limb weakness OR arm weakness OR leg weakness OR atrophy OR paraplegia OR paresis OR spastic paraplegia OR spastic paresis OR paralysis
21	#1 OR #2
22	#3 OR #4

23	#5 OR #6 OR #7 OR #8 OR #9 OR #10 OR #11 OR #12 OR #13 OR #14 OR #15 OR #16 OR #19 OR #20
24	#17 OR #18
25	#21 AND #22 AND #23 AND #24

Google Scholar

Spasticity AND "botulinum toxin" AND strength

Ovid MEDLINE ALL <1946 to March 19, 2024>

1	exp Cerebrovascular Disorders/
2	neurological disorder.mp. or exp Nervous System Diseases/
3	multiple sclerosis.mp. or exp Multiple Sclerosis/
4	cerebral palsy.mp. or exp Cerebral Palsy/
5	Stroke/ or stroke, lacunar/ or subarachnoid h*morrhage/ or subarachnoid h*morrhage/ or subdural h*morrhage/ or subdural h*matoma/ or cerebrovascular disorders*/ or h*morrhage/ or intracranial h*morrhage/ or intracranial thrombosis/ or intracranial embolism/ or brain hypoxia/ or brain isch*mia/ or cerebral h*morrhage/ or intracranial embolism/ or intracranial thrombosis/ or intracranial arterial diseases/ or cerebral arterial diseases/ or carotid artery diseases/ or basal ganglia cerebrovascular disease/ or cerebral isch*mia/ or intracranial sinus thrombosis/ or cerebrovascular accident/ or CVA/ or cerebral infarct*/ or brain infarct*/ or intracranial h*morr*/ or intra-cranial h*morr*/ or intra-cranial h*morr*/ or cerebral haemorr*/ or cortical haemorr*/ or cortical ischaem*/ or cerebral ischaem*/ or cerebral ischem*/ or brain ischaem*/ or cerebrovasc* disord*/ or cerebral aneurysm*/ or brain aneurysm*/ or hypoxic brain injur*/ or anoxic brain injur*/ or subdural haem*/ or subdural H*m*/ or intracranial thromb*/ or intra-cranial thomb*/ or intra-cranial emb*/ or intracranial emb*/ or brain lesion*/ or cerebral lesion*/ or cortical lesion*/ or cerebrovascular disease/ or basal ganglia h*morrhage/ or brain isch*mia/ or brain infarction/ or brain stem infarctions/ or lateral medullary syndrome/ or infarction, anterior cerebral artery/ or infarction, middle cerebral artery/ or infarction, posterior cerebral artery/ or hypoxia-ischemia, brain/ or carotid artery diseases/ or carotid artery thrombosis/ or arteriovenous malformations/ or intracranial arteriovenous malformations/ or putaminal h*morrhage/ or putaminal h*morrhage/ or poststroke/ or post-stroke/ or apoplex*/ or cerebral vasc*/ or cerebralvasc*/ or cva/ or SAH/ or cerebr*/ or cerebell*/ or vertebrobasilar artery/ or intracerebral/ or intracran*/ or intra-cranial/ or basal gangli*/ or hemisphere*/ or subarachnoid/ or arteriovenous/ or arterio-venous/ or brain tumour/ or brain tumor/
6	brain injury.mp. or exp Brain Injuries/
7	exp traumatic brain injury/ or exp brain injury/ or TBI/ or ABI/ or Acquired brain injur*/ or traumatic brain injur*/ or head injur*/ or acquired brain injur*/ or brain stem injur*/ or cerebell* injury/ or diffuse axonal injur*/ or brain h*morrhage/ or brain hypoxia/ or brain cortex lesion/ or brain cyst/ or brain damage/ or brain cancer/ or brain tumour/ or cerebrovascular trauma/ or cerebral trauma/ or cerebral damage.mp. [mp=title, abstract, original title, name of substance word, subject heading word, floating sub-heading word, keyword heading word, organism supplementary concept word, protocol supplementary concept word, rare disease supplementary concept word, unique identifier, synonyms]
8	exp cerebrovascular accident/ or exp cerebrovascular disease/ or exp cerebrovascular disorders/ or exp brain ischemia/ or cerebrovasc.mp. or exp infarction/ or exp cerebrovascular accident/
9	exp paresis/ or Hemiparesis/ or hemiplegia/ or hemipleg*/ or hemipar*/ or paralysis/ or paraparesis/ or mono paresis/ or exp muscle weakness/ or exp limb weakness/ or exp arm weakness/ or exp weakness/ or exp leg weakness/ or atrophy/ or paraplegia/ or paresis/ or spastic paraplegia/ or spastic paresis/ or paralysis/
10	exp muscle, skeletal/ or exp grip strength/ or exp pinch strength/ or exp muscle strength/ or exp hand strength/ or strength.mp.
11	muscle contraction/ or eccentric contraction/ or concentric contraction/ or isometric contraction/ or isotonic contraction/ or isokinetic contraction/ or muscle contraction/ or Muscle weakness/ or weakness/ or paraparesis/ or paresis/ or strength/ or muscle strength/ or hand strength/ or pinch strength/ or motor control/ or strength/ or motricity index/ or dynamo*/ or Manual muscle test/ or Musc* power/ or force generation/ or force production/ or force.mp. or (muscle* adj4 (contraction* or voluntary activation or force* or power)).mp. or maximal voluntary contraction*.mp.
12	exp muscle spasticity/ or muscle hypertonia/ or muscle rigidity/ or muscle tonus/ or spasm/ or dystonia/ or paraparesis/ or spastic*/ or hypertonicity/ or muscle overactivity/ or upper motor neuron* syndrome/ or stretch reflex/ or hypertonia/ or muscle tone/ or muscle spasm/ or paraplegia/ or hyperton*/ or spastic paresis/ or spasticity/ or myotatic reflex/ or stretch reflex/
13	spasticity/ or exp spastic paresis/ or exp muscle hypertonia/ or exp muscle rigidity/
14	exp Botulinum Toxins, Type A/ or exp Botulinum Toxins/ or Botulinum/ or botulinum toxin/ or BTX-A/ or BTXA/ or BoNT-A/ or BONTA/ or disport/ or Xeomin/ or AbobotulinumtoxinA/ or OnabotulinumtoxinA/ or IncobotulinumtoxinA/ or botulinum toxin.mp. or exp botulinum toxin/ or exp botulinum toxin A/
15	1 or 2 or 3 or 4 or 5 or 6 or 7 or 8 or 9
16	9 or 10 or 11

17	12 or 13
18	14 and 15 and 16 and 17

PEDro

Search strategy: Advanced
Abstract and Title: 1st Search: “spas*” AND “bot*” AND “stren*”
Abstract and Title: 2nd Search: “spas*” AND “bot*” AND “weak*”
Abstract and Title: 3rd Search: “spas*” AND “bot*” AND “musc*”

Pubmed <1984 - March 19th 2024>

(((((((((((nervous system diseases) OR (neurological disorder OR neurological disorder*)) OR (brain injury OR brain injur* OR head injur* OR acquired brain injur* OR brain damage OR brain stem injur* OR cerebrovascular accident* OR cerebell* injury OR diffuse axonal injur* OR brain haemorrhage OR brain hypoxia OR brain cortex lesion OR brain cyst OR brain damage OR brain cancer OR brain tumour OR cerebrovascular trauma OR cerebral trauma OR cerebral damage OR TBI OR ABI OR Acquired brain injur* OR traumatic brain injur*)) OR (multiple sclerosis)) OR (cerebral palsy)) OR (paresis OR hemiparesis OR hemiplegia OR hemipleg* OR hemipar* OR paralysis OR paraparesis OR mono paresis OR muscle weakness OR limb weakness OR arm weakness OR leg weakness OR atrophy OR paraplegia OR paresis OR spastic paraplegia OR spastic paresis OR paralysis)) OR (Stroke OR lacunar stroke OR subarachnoid haemorrhage OR subdural haemorrhage OR subdural haematoma OR cerebrovascular disorders* OR haemorrhage OR intracranial haemorrhage OR intracranial thrombosis OR intracranial embolism OR brain hypoxia OR brain ischemia OR cerebral haemorrhage OR intracranial embolism OR intracranial thrombosis OR intracranial arterial diseases OR cerebral arterial diseases OR carotid artery diseases OR basal ganglia cerebrovascular disease OR cerebral ischemia OR cerebral ischemia OR intracranial sinus thrombosis OR cerebrovascular accident OR CVA OR cerebral infarct* OR brain infarct* OR intracranial haemorr* OR intra-cranial haemorr* OR intracranial hemorr* OR intra-cranial hemorr* OR cerebral haemorr* OR cortical haemorr* OR cortical ischaem* OR cerebral ischaem* OR cerebral ischem* OR brain ischaem* OR cerebrovasc* disord* OR cerebral aneurysm* OR brain aneurysm* OR hypoxic brain injur* OR anoxic brain injur* OR subdural haem* OR subdural Hemo* OR intracranial thromb* OR intra-cranial thomb* OR intra-cranial embo* OR intracranial embo* OR brain lesion* OR cerebral lesion* OR cortical lesion* OR cerebrovascular disease OR basal ganglia cerebrovascular disease OR basal ganglia h?morrhage OR brain ischemia OR brain infarction OR brain stem infarctions OR lateral medullary syndrome OR cerebral infarction OR anterior cerebral artery infarction OR middle cerebral artery infarction OR posterior cerebral artery infarction OR brain hypoxia-ischemia OR carotid artery diseases OR carotid artery thrombosis OR arteriovenous malformations OR intracranial arteriovenous malformations OR putaminal hemorrhage OR putaminal haemorrhage OR poststroke OR post-stroke OR apoplex* OR cerebral vasc* OR cerebralvasc* OR cva OR SAH OR cerebr* OR cerebell* OR vertebrobasilar artery OR intracerebral OR intracran* OR intra-cranial OR basal gangli* OR hemisphere* OR subarachnoid OR arteriovenous OR arterio-venous OR brain tumour OR brain tumor)) OR incomplete spinal cord inju* AND ((paresis OR hemiparesis OR hemiplegia OR hemipleg* OR hemipar* OR paralysis OR paraparesis OR mono paresis OR muscle weakness OR limb weakness OR arm weakness OR leg weakness OR atrophy OR paraplegia OR paresis OR spastic paraplegia OR spastic paresis OR paralysis) OR (muscle strength OR muscle contraction OR eccentric contraction OR concentric contraction OR isometric contraction OR isotonic contraction OR isokinetic contraction OR muscle contraction OR Muscle weakness OR weakness OR paraparesis OR paresis OR strength OR muscle strength OR hand strength OR pinch strength OR motor control OR strength OR motricity index OR dynamo* OR Manual muscle test OR Musc* power OR force generation OR force production OR force OR torque OR (muscle* adj4 (contraction* or voluntary activation or force* or power)) OR maximal voluntary contraction*))) AND (muscle spasticity OR muscle hypertonia OR muscle rigidity OR muscle tonus OR spasm OR dystonia OR paraparesis OR spastic* OR hypertonicity OR muscle overactivity OR upper motor neuron* syndrome OR stretch reflex OR hypertonia OR muscle tone OR muscle spasm OR paraplegia OR hyperton* OR spastic paresis OR spasticity OR myotatic reflex OR stretch reflex)) AND (botulinum OR botulinum toxin OR BTX-A OR BTXA OR BoNT-A OR BONTA OR dysport OR Xeomin OR AbobotulinumtoxinA OR OnabotulinumtoxinA OR IncobotulinumtoxinA))

Web of Science <1900 - March 19th 2024>

1	Stroke OR h$morr* OR cerebr$vascular accident* OR CVA OR cerebral infarct* OR brain infarct* OR isch$m* OR cerebrovasc* disord* OR brain lesion* OR cerebral lesion* OR cortical lesion* OR cerebell* OR multiple sclerosis OR Cerebral palsy OR brain injur*
2	hemipleg* OR hemipar* OR paralysis OR paraparesis OR muscle weakness OR limb weakness OR arm weakness OR leg weakness OR atrophy OR paraplegia OR paresis
3	contraction OR "muscle contraction" OR "muscle weakness " OR weakness OR strength OR motor control OR motricity index OR dynamo* OR "manual muscle test*" OR musc* power OR force
4	spasticity OR muscle rigidity OR muscle ton* OR spasm OR dystonia OR spastic* OR hyperton* OR muscle overactivity OR upper motor neuron* syndrome OR myotatic reflex OR stretch reflex

5	botulinum OR botulinum toxin OR BTX-A OR BTXA OR BoNT-A OR BONTA OR dysport OR Xeomin OR AbobotulinumtoxinA OR OnabotulinumtoxinA OR IncobotulinumtoxinA
6	#5 OR #4 AND #3 AND #2 AND #1
