# Supplementary material for: The Effect of Botulinum Neurotoxin-A (BoNT-A) on Muscle Strength in Adult-Onset Neurological Conditions with Focal Muscle Spasticity: A Systematic Review
Source: Toxins (Basel). 2024 Aug 8;16(8):347. doi: 10.3390/toxins16080347 (PMC11359732; doi:10.3390/toxins16080347)
Supplement: Supplementary file 1 [file toxins-16-00347-s001.zip › Supplementary Table S7. The methodological quality modified downs and black and Pedro scores - Revised.pdf]

Supplementary Table S7. Methodological quality assessment modified Downs and Black checklist and PEDro scale (n = 54).

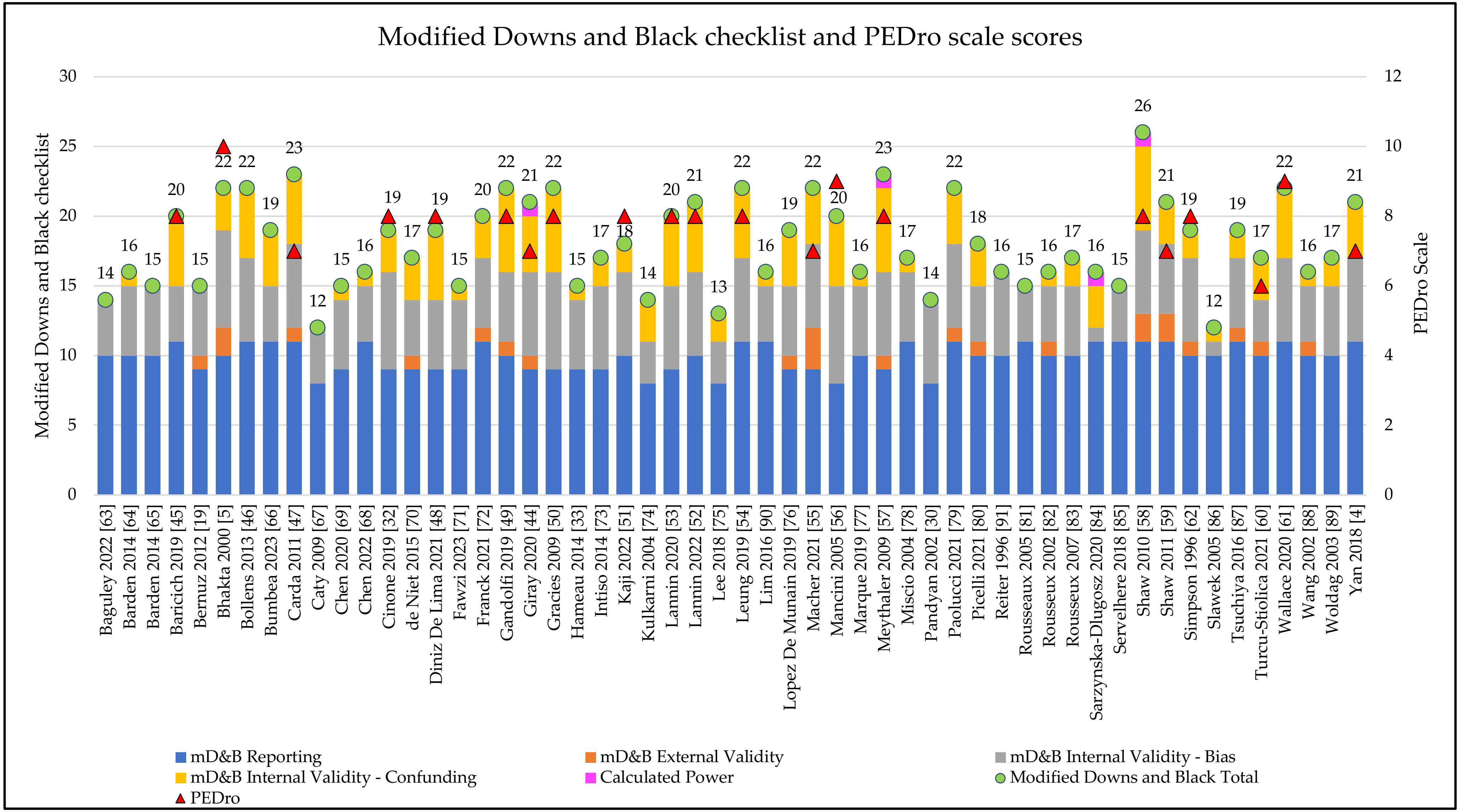

Supplementary File 7. The methodological quality of the Modified Downs and Black checklist and PEDro scale
